# Psychotropic and Opioid-Based Medication Use among Economically Disadvantaged African-American Older Adults

**DOI:** 10.3390/pharmacy8020074

**Published:** 2020-04-27

**Authors:** Mohsen Bazargan, Sharon Cobb, Cheryl Wisseh, Shervin Assari

**Affiliations:** 1 Department of Family Medicine, College of Medicine, Charles R Drew University of Medicine and Science, Los Angeles, CA 90059, USA; Mohsenbazargan@cdrewu.edu (M.B.); cherylwisseh@cdrewu.edu (C.W.); 2 Department of Family Medicine, University of California, Los Angeles (UCLA), Los Angeles, CA 90095, USA; 3 School of Nursing, Charles R Drew University of Medicine and Science, Los Angeles, CA 90059, USA; sharoncobb@cdrewu.edu; 4 Department of Pharmacy Practice, West Coast University School of Pharmacy, Los Angeles, CA 90004, USA

**Keywords:** opioids, psychotropic medications, pain, disparities, pain-killers

## Abstract

African-American older adults, particularly those who live in economically deprived areas, are less likely to receive pain and psychotropic medications, compared to Whites. This study explored the link between social, behavioral, and health correlates of pain and psychotropic medication use in a sample of economically disadvantaged African-American older adults. This community-based study recruited 740 African-American older adults who were 55+ yeas-old in economically disadvantaged areas of South Los Angeles. Opioid-based and psychotropic medications were the outcome variables. Gender, age, living arrangement, socioeconomic status (educational attainment and financial strain), continuity of medical care, health management organization membership, sleeping disorder/insomnia, arthritis, back pain, pain severity, self-rated health, depressive symptoms, and major chronic conditions were the explanatory variables. Logistic regression was used for data analyses. Arthritis, back pain, severe pain, and poor self-rated health were associated with opioid-based medications. Pain severity and depressive symptoms were correlated with psychotropic medication. Among African-American older adults, arthritis, back pain, poor self-rated health, and severe pain increase the chance of opioid-based and psychotropic medication. Future research should test factors that can reduce inappropriate and appropriate use and prescription of opioid-based and psychotropic medication among economically disadvantaged African-American older adults.

## 1. Introduction

Management of chronic health conditions with opioid-based and psychotropic medications can be complex. Recent guidelines by the American Geriatric Society have recommended that health providers begin treatment of chronic pain with low doses of opioids among older adults, with careful monitoring [1]. This course of treatment is also suggested for individuals with persistent chronic pain who report no relief from non-opioid medication and experiencing functional impairment or lower quality of life [2]. Even though opioid-based medications are commonly provided to older adults for the management of their chronic pain conditions, there is a lack of strong evidence supporting these recommendations in older adults, particularly those with multiple comorbidities [3]. Common comorbidities in older adults may either increase the risk of side effects from opioid use or attenuate the benefits [3].

Multiple studies have shown that African-American older adults with experience chronic and intense pain may be untreated or under-treated for their pain [4,5,6]. A review of current literature clearly shows a disparity in the management of pain among older African-American adults [7]. In fact, inadequately treated pain can interfere with daily functional activities and cause depression [8]. However, data from state-certified addiction treatment centers show that the proportion of older African-American adults seeking treatment for opioid use disorder rose steadily between 2004–2015 [9]. Thus, African-American adults are less likely to be prescribed opioid-based medications for the treatment of severe pain compared to non-Hispanic Whites; yet at the same time, they are more likely to seek treatment for opioid use disorders than their White counterparts. Furthermore, opioid-related mortalities are sharply increasing among African Americans, exceeding the rates of Whites in some regions of the United States [10,11]. A recent study of opioid intoxications utilizing the emergency department revealed that African-Americans had higher chronic disease morbidity compared to Whites [12]. Taken together, these pieces of evidence point to mismanagement of pain among older African-American adults.

Psychotropic medications are frequently prescribed for older adults to manage behavior, sleeping disorders, pain, and psychiatric symptoms. Yet older adults may experience adverse reactions with psychotropic medication use, attributed to age-related changes to pharmacological processes and dangerous side effects [13]. Both the National Ambulatory Medical Care Survey and Medicare claims show that the prevalence of psychotropic prescription medications use among older adults substantially increased within the last decades [14,15]. However, these data show that racial and ethnic minority Medicare beneficiaries had higher odds of use of potentially inappropriate psychotropic prescriptions [14]. Even though studies investigating these two forms of medications among African Americans are almost nonexistent, one particular study found that 59% of 100 African American young adults used psychotropic medications with 3,4-Methylenedioxy methamphetamine (MDMA) drugs, compared to 35% who used opioids and MDMA [16]. This may explain that inappropriate medication use may require further study within this population. Yet, the combination of psychotropic medication and opioids may be more concerning due to its occurrence in older adults and its effects. Additionally, there are several major concerns regarding the inappropriate use of psychotropic medications among older adults, including strong evidence of an association between these medications and the risk of falls [17].

African Americans are largely untreated or undertreated for mental health symptoms compared to White counterparts [18,19,20]. Studies have shown that African Americans with depression are less likely to have prescribed antidepressants or anxiolytic treatment for their mental symptoms [19,21], but have higher rates of psychotropic prescriptions than Whites [22,23]. Prescriptions for psychotropics were very common for adults with mental illness until 2006 when a sharp decline occurred after the Food and Drug Administration reported concern about the adverse effects of antipsychotics among the elderly population. These side effects among older adults may include hepatic failure, drug abuse and dependency, and increased mortality [24]. However, African-American older adults may still be receiving prescriptions for psychotropic medications with adverse effects.

Based on the data from the 2004–2013 National Ambulatory Medical Care Survey, concurrent use of opioid-based and psychotropic medications largely contributes to polypharmacy and inappropriate medication among older adults. Concurrent use of opioid-based and psychotropic medication use was documented at an estimated 1.5 million health care encounters each year between 2011 and 2013 [25]. Another recent study among older adults insured by the AARP Medicare supplement plan, among those who used opioid-based medications, 57% used opioids only, 28% used opioids plus 1 additional central nervous system medication, and 15% used ≥2 additional medications [26]. A review of current literature shows that most often, the combination of opioid-based and psychotropic medication is prescribed to older adults in order to manage anxiety, insomnia, or depression associated with their chronic pain [26]. Finally, the National Hospital Ambulatory Medical Care Survey (2005–2015) showed that despite guidelines advising against concurrent use of psychotropic medication and opioids, older adults still report high co-prescription rates of the two medication classes [27]. 

Even though there are adverse effects of concurrent use of opioids and psychotropic medications, there may be various effects that may present separately within this population. Members of this group residing in economically underserved areas, such as South Los Angeles in California, maybe at risk for multiple health disparities. Older African Americans residing in this area have a high rate of presenting to the emergency department [28] as well as underestimate their risk for cancer [29]. Due to this group being undertreated or untreated for specific issues, such as pain, it is plausible to expect high levels of under-treatment of pain and depression in this population.

## 2. Aims

The purpose of this study was to compare opioid-based and psychotropic medication use and pain among older African-American adults residing in South Los Angeles. To better understand the prevalence and impact of medication use, we aimed to examine correlates associated with opioid-based medication and psychotropic medication use separately, with major emphasis on chronic pain, mental health status, chronic comorbidities, and social demographics. This is important as this target population of underserved older adults may be at high risk for adverse effects related to medication usage. 

## 3. Materials and Methods

### 3.1. Design and Setting

This was a cross-sectional survey that targeted African-American older adults residing in South Los Angeles. Fully structured and face to face interviews were collected between 2015 and 2018. All participants provided written informed consent prior to enrollment in the study. This community-based study used non-random sampling and recruited 740 African-American adults aged 65 years and older. The study protocol received ethical approval by the Charles R. Drew University of Medicine and Science (CDU) Institutional Review Board (IRB), Los Angeles, California, USA (CDU IRB#: 14-12-2450-05). All participants provided a written informed consent before they were enrolled to the study. Participants were financially compensated.

### 3.2. Participants

The recruitment site for this study was South Los Angeles, Service Planning Area 6 (SPA 6) of Los Angeles County. SPA 6, which includes South Los Angeles, serves multiple cities and unincorporated areas, including the communities of Athens, Florence, Compton, Crenshaw, Paramount, Lynwood, Hyde Park, and Watts. Together, SPA 6 is comprised of over one million adults [30]. Currently, SPA 6 is one of the most underserved and under-resourced areas in the nation, with 28% identifying as African Americans and 68% as Hispanic. Participants were eligible if they identified as African American or Black, were 65 years or older, reported at least one cardiometabolic disease, and could independently complete a full interview in English. Compared to the rest of Los Angeles County, residents of South Los Angeles SPA 6 are disproportionately affected by health disparities [31]. For example, the age-adjusted diabetes death rate in South Los Angeles is almost five times higher than in West Los Angeles [31].

### 3.3. Measurements

Prior to this study, in-depth cognitive interviews were conducted with a sample of 10 potential participants to assess the content validity of survey instruments. Following, the survey instruments were pilot tested with a sample of 21 older adults, and minor modification was made.

#### 3.3.1. Sociodemographic Characteristics

The following variables were examined: gender, age, educational attainment, and living arrangement. Educational attainment was operationalized as an interval variable (years of education). A higher number indicated more years of educational attainment.

#### 3.3.2. Financial Strain

To capture the impact of economic burden, the financial strain was assessed. Within SPA 6, almost 34% have household incomes below the federal poverty level [30]. Additionally, the study’s setting has experienced the largest increase in overweight and obesity in the state of California within the past decade [32]. Five items were used to measure participants’ financial stress. Respondents were asked if they were able to purchase the following in the previous 12 months: (1) food for themselves and family, (2) clothes for themselves and family, (3) pay rent or mortgage, (4) monthly bills, and (5) make ends meet. A total "financial difficulty" score was calculated, with the average score of five items, ranging from 1 to 5. Greater financial strain was observed with higher scores on items. This measurement of financial strain was in line with the measurement of financial difficulties among low-income individuals by Pearlin [33] (Cronbach’s alpha = 0.92).

#### 3.3.3. Health Maintenance Organization (HMO) Membership

HMO membership was assessed by asking participants where they usually receive their medical care.

#### 3.3.4. Self-Rated Health (SRH)

A standard item was used to measure self-perceived health status. Participants were asked the following single question: “In general, would you say your health is (1) Excellent; (2) Very good; (3) Good; (4) Fair; and (5) Poor?” This measure is commonly used in national surveys [34,35].

#### 3.3.5. Medication Use Inventory

The drug inventory method was used to assess the use of both prescribed, over the counter, and non-prescribed medications. This instrument focused only on prescribing and dosage instructions and did not assess individual-reported use. All medications taken by participants within the two weeks prior to the interviews were inspected. The research team collected the following medication data: generic/brand name of the medication, the strength of the drug, date medication was filled, the number of refills, expiration data, instructions, special warnings, as well as the name and contact number of providers and pharmacy that prescription was filled. 

#### 3.3.6. Severity of Pain

Pain was measured using the four subscales outlined in the Short-Form McGill Pain Questionnaire-2 (SF-MPQ-2) [36,37]. Participants self-reported the level to which they experienced each of the four subscales, which is composed of 22 pain items in the past week using an 11-point numeric rating scale (0 = “none” to 10 = "worst possible"). In particular, the SF-MPQ-2 is composed of (1) continuous pain (throbbing, cramping, gnawing, aching, heavy, and tender pain); (2) intermittent pain (shooting, stabbing, sharp, splitting, electric-shock, and piercing pain); (3) predominately neuropathic pain (numbness, cold-freezing, itching, tingling or ‘pins and needles,’ light touch, and hot-burning); and 4) affective descriptors pain (tiring-exhausting, sickening, fearful, and punishing-cruel pain). Severe pain was defined according to the World Health Organization (WHO) scale, which was 7 to 10 on the 11-point numeric rating scale mentioned above [38]. An average pain score was computed for each of the four pain subscales [36,37]. Following, all scores on the SF-MPQ-2 were averaged, resulting in a total score of the assessment of pain [36,39]. This assessment tool has excellent reliability and validity as an index for measuring pain among older adults and has been extensively used in the community, acute and long-term care settings [40,41].

#### 3.3.7. Self-Reported Medical Conditions

Participants were asked to report major chronic conditions they were currently managing by acknowledging “Yes” or “No” to the condition. The major diagnoses included hypertension, diabetes, stroke, cardiac issues/heart problems, asthma, cancer, and gastrointestinal conditions. The number of chronic conditions was tabulated for participants. The variable was operationalized as a continuous variable (higher score meant more conditions).

In addition, participants asked to report if they had been diagnosed with other medical conditions, specifically arthritis, back pain, migraine headache, and sleep disorder. These conditions were treated as dichotomous variables (yes/no) for this study. 

### 3.4. Data Analysis

Our analysis had three parts. The first part was a descriptive analysis of all participants, which included measurement of categorical variables (frequencies and percentages) and continuous measures (means and standard deviations). Then we ran chi-square and independent t-test to compare participants based on the use of opioid-based and psychotropic medications. Multivariate logistic regression was performed to estimate independent correlates of using prescribed opioid-based and psychotropic medications. A *p*-value of less than 0.05 was considered significant. 

## 4. Results

Table 1 reports the characteristics of the study sample. This study included 740 African-American individuals aged 55 years and older (mean, 71.7 ± 8.4). More than 64% of the participants were women. About 50% of the sample reported living alone. More than 35% of our sample reported had a high school diploma, and 25% reported 0-11 years of education. More than one-third (35.4%) of participants indicated that they received their medical care from a health maintenance organization.

### 4.1. Psychotropic and Opioid-Based Medication Use

Examination of medication containers and face-to-face interviews show that 198 (27.7%) of participants used opioid-based or psychotropic medication. Almost 16% (n = 113) and 16.3% (n = 116) of participants used at least one psychotropic and opioid-based medications respectively. Of those who use opioid-based medications, 73% used opioids only, 28% used opioids plus at least one psychotropic medication. Of 116 participants who used opioid-based medications, close to 9% used more than one. Two of the most commonly used opioid-based medications among our sample were hydrocodone-acetaminophen (24.7%) and tramadol (22.7%). The average number of prescription drugs used among opioid-based medication users and non-users is 8.28 (SD: 3.2) and 5.41 (SD: 3.3), respectively.

One out of four participants who used psychotropic medications took at least two types of psychotropic medication. The most frequently used psychotropic medications were gabapentin (16.5%), sertraline (6.2%), and amitriptyline (4.6%). The average number of prescription drugs used among psychotropic medication users and non-users was 8.10 (SD: 3.7) and 5.48 (SD: 3.0), respectively.

### 4.2. Bivariate Associations

Table 2 shows the bivariate correlation between the independent variables and medication use. At the bivariate level, women, participants with poorer self-rated health, those with sleeping disorder/insomnia, those diagnosed with arthritis and back pain, participants with a higher level of pain and depressive symptoms as well as those with a higher number of major chronic conditions were more likely to use opioid-based medications. However, younger participants, those with a higher level of financial strain, those who reported poorer health, those with sleeping disorder/insomnia, those diagnosed with arthritis, migraine, and back pain, participants with a higher level of general pain and with a higher number of depressive symptoms and a higher number of major chronic conditions were more likely to use psychotropic medications.

### 4.3. Multivariate Logistic Regression

Controlling for all other variables, four independent variables showed a statistically significant correlation with opioid-based medication use. They are self-rated health status, being diagnosed with arthritis, back pain, and general level of pain. Table 3 (first three columns) shows that participants who reported poorer levels of health were 1.3 (95% CI: 1.016–1.680, *p* < 0.05) times more likely to use opioid-based medications. Additionally, participants with back pain and arthritis were 2.206 (95% CI: 1.338–3.638, *p* < 0.01) and 2.087 (95% CI: 1.148–3.795, *p* < 0.05) times more likely to use opioid-based medications, respectively. Finally, a unit increase in general pain (with a scale of 0–10) increased the likelihood of using opioid-based medication by 12% (OR: 1.122; 95% CI: 1.006–1.251; *p* < 0.05).

Controlling for all other relevant variables, only depressive symptoms, and level of pain were statistically associated with psychotropic medication use among our sample of underserved African-American older adults. An additional point increase in the depressive symptoms (scale of 0–14) increased the likelihood of the use of psychotropic medications by more than 14% (OR: 1.143; 95% CI: 1.053–1.241; *p* < 0.001). Similarly, a unit increase in general pain (with a scale of 0–10) increased the likelihood of using psychotropic medication by 12% (OR: 1.126; 95% CI: 1.008–1.258; *p* < 0.05).

## 5. Discussion

Almost 30% of participants used psychotropic or opioid-based medications, with 16% using psychotropic medications and 16% using opioid-based medications. At least 25% of psychotropic medication users were taking two classes of psychotropic drugs. The following factors were predictors of opioid-based medication use: self-rated health status, arthritis, back pain, and overall level of pain. With regard to psychotropic medication use, depressive symptoms and level of pain were found to be predictors.

Higher levels of pain were significantly related to psychotropic medication use. Similar studies support this finding with users of psychotropic drugs reporting poor pain control [42,43]. Helstrom and colleagues (2018) found that chronic pain was identified in 14% of 250 older adults who were recently prescribed a psychotropic medication, who also reported pain interference with daily activities [44]. It is important to note that pain is often a symptom of mental illness, such as depression, and mentally ill persons may be untreated for their pain due to the priority given to treating symptoms of mental illness [45]. Individuals may receive antipsychotic medication prescriptions from their mental health care provider or psychiatrist, which may neglect to assess and treat chronic pain effectively. Coordinated care is required for these individuals as treatment of pain may be required from their general health care provider or pain management specialists [46]. This population may benefit from both pharmacological and non-pharmacological sources of pain management.

Various types of pain were a significant predictor of opioid medication use, which is not surprising. Yazdanshenas, Bazargan, and colleagues (2016) revealed that there is severe mismanagement of pain among African-American older adults, which may be attributed to potentially inappropriate medications, such as opioids [47]. Further, Burgess and colleagues (2016) found opioid users reported greater pain interference compared to non-opioid users [48]. Even though African-Americans are undertreated with opioid-based medications, they are at higher odds for opioid abuse and dependence as opposed to Whites [49]. African Americans’ adherence to pain medication may be decreased, and adherence to opioids may be worsened with around-the-clock use [50]. Opioids may provide short-term relief for arthritic and back pain, but long-term efficacy is unknown, with greater potential for dependency and misuse. Recent approaches to assist opioid users to withdraw from opioids and start medication-assisted treatment may be appropriate for African-American older adults using opioids.

Depressive symptoms were also a major predictor of psychotropic medication use. A common use of psychotropic medications is to treat or alleviate mental symptoms, which includes depressive symptoms. Antipsychotic medications have been shown to be effective for depressive symptoms among African Americans [51,52], yet they have lower odds of being prescribed and adherent compared to Whites [19,53]. Interventions focused on psychotropic medication use among African-American older adults should center on raising awareness of the potential adverse effects of these medications and recommending less harmful, newer generations of antipsychotics.

Individuals using opioid-based medications are more likely to have poorer self-rated health. This finding is supported in the literature among African Americans [54]. Further, poor health has been found to be a significant factor in prescription opioid misuse among African Americans [55]. Other populations groups have also expressed lower self-rated health scores with opioid use [56]. Even though only a bivariate correlation was found, African Americans using antipsychotics were found to have a lower quality of life, a finding corroborated by other studies [57,58]. Low quality of life has been documented as prevalent among African Americans [59,60,61,62]. Harrison and colleagues (2018) found that an increased number of psychotropic medications in individuals was associated with poorer self-rated health and quality of life [63].

Among this sample, multiple chronic conditions are also related to opioid-based and psychotropic medication use. African-American older adults may present with high rates of mental illnesses associated with age, such as vascular depression, which may be untreated [64]. Opioid use has been associated with multiple chronic conditions, such as cancer [65], musculoskeletal pain [66], and post-traumatic stress disorder [67], whereas psychotropic drug use is associated with various illnesses, such as Alzheimer’s Disease [68] and epilepsy [69]. Due to the various conditions that individuals may have, careful assessment of all chronic illnesses and conditions should occur due to potential interactions and complex health history.

A significant relationship was found between sleep disorders and opioid-based medications. Liu and colleagues found that concurrent insomnia and depressive symptoms increased the prescription supply of both opioids and psychotropic medications [70]. Even though they were not significant in multivariate regression results, sleep disorders may be a contributing factor to opioid medication use and should be carefully examined by health providers [70], especially since opioid dependency may increase if sleep issues are present.

## 6. Implications

Very recently, the United States faced a national opioid crisis that cried out for potential solutions. Although not a crisis of the same size, psychotropic medications can also have lasting harmful effects. As individuals increase their use of these types of medications, they also increase their mortality risk [71]. The value of this study is that we have isolated several risk factors that can be used by health providers to identify those who are at greater risk. We found that pain, sleep disorders, and depressive symptoms are conditions that providers are likely to treat by prescribing antipsychotic or opioid-based medications [71]. Further research is warranted for this population who may be at risk for medication-related challenges such as nonadherence, inappropriateness, or misuse [72]. Interventions ought to be put in place to address the misuse of these medications among African-American older adults. For example, screening instruments for predicting misuse and dependency of both opioid and antipsychotic medication should be utilized in clinical practice. Medication-assisted treatment to reduce dependence on these medications should be provided to underserved African-American older adults using opioids. Hansen and colleagues (2016) found that medication-assisted treatment was more common in higher-income areas and least common among African Americans living in impoverished communities [73]. In addition, providers should focus on education and outreach efforts aimed at increasing medication knowledge and treatment plan effectiveness among African-American older adults.

## 7. Limitations

This study has a few methodological limitations. Due to this study’s cross-sectional nature, causal inferences are not recommended. As we utilized consecutive sampling, the generalizability of the findings is also limited. In addition, we did not have access to medical records and relied on self-reporting of chronic conditions, which may lead to measurement bias. Third, we did not assess the length of opioid and/or antipsychotic use. We also did not study adherence or self-reported use of prescribed medications. Additionally, depressive symptoms may have been underreported due to cultural stigma within the African American population toward mental illness [74,75,76]. This may lead to individuals not wanting to acknowledge or being unable to recognize signs of depression. Finally, we did not have access to medical records and charts, so we could not determine unnecessary prescriptions. Despite these limitations, this study contributes to the current knowledge of psychotropic and opioid-based medication use among African-American older adults.

## 8. Conclusions

This study highlights factors associated with use of psychotropic and opioid-based medications. A major factor was patients who are experiencing severe pain, back pain, and poor self-rated health. This finding emphasizes the importance of effective pain management among this population of African-American older adults, who may be experiencing poor pain control. Furthermore, improved coordination of care and newer approaches to managed care are necessary to assist this population in improving their mental health and wellness. Analyzing the effects of the duration of these medications and its association with mental health may also serve to highlight opportunities for intervention into poorer health outcomes amongst this population.

## Figures and Tables

**Table 1:** Characteristics of middle-aged underserved African-American adults (n = 740)**.**

	n	%
***Gender***		
Male	266	35.9
Female	474	64.1
***Age***		
55–64	120	16.2
65–74	360	48.6
≥ 75	260	35.1
***Education***		
No high school diploma	183	24.7
High school diploma	265	35.8
Some college or college degree	292	39.5
***Living Alone***		
No	294	39.7
Yes	446	60.3
***HMO Status***		
No	478	64.6
Yes	262	35.4
**Sleeping disorder/Insomnia**		
No	541	73.3
Yes	197	26.7
**Arthritis**		
No	248	33.6
Yes	490	66.4
**Migraine Headache**		
No	597	80.9
Yes	141	19.1
**Back pain**		
No	385	52.2
Yes	353	47.8
		Mean ± SD
***Age*** *(years: 55–96)*		*71.73 ± 8.36*
***Education Attainment*** *(1–16)*		*12.74 ± 2.24*
***Financial Strains*** *(1: None–5: High)*		*1.83 ± 1.13*
***Level of Pain*** *(0: Low–10: High)*		*2.03 ± 2.25*
***Continuity of Care*** *(0: Low to 3: High)*		*2.49 ± 0.66*
***Self-rated Health*** *(1: Excellent–5: Poor)*		*2.19 ± 1.34*
***Depressive Symptoms*** *(0: None–14: Severe)*		*2.47 ± 2.77*
***Seven Major Chronic Conditions*** *(0–7)*		*2.42 ± 1.31*

**Table 2:** Bi-variate correlates of opioid-based and psychotropic medication use among underserved middle-aged and older African-American adults (n = 740)**.**

Independent Variables	Opioid-Based Medications		*p*	Psychotropic Medications		*p*
	NO	Yes		NO	Yes	
	N (%) or (X ± SD)	N (%) or (X ± SD)		N (%) or (X ± SD)	N (%) or (X ± SD)	
**Gender**						
Male	**223 (85)**	**29 (12)**	**0.01**	218 (86)	36 (14)	0.368
Female	**372 (81)**	**87 (19)**		383 (83)	77 (17)	
**Age**	(71.8 ± 8.21)	(72.3 ± 8.44)	0.556	**(72.2 ± 8.24)**	**(70.0 ± 7.94)**	**0.009**
**Education**	(12.7 ± 2.22)	(12.6 ± 2.28)	0.446	(12.7 ± 2.27)	(12.8 ± 2.00)	0.676
**Financial Strains**	(1.78 ± 1.09)	(1.84 ± 1.14)	0.632	**(1.70 ± 1.06)**	**(2.29 ± 1.16)**	**0.000**
**Living Alone**						
No	245 (86)	39 (14)	0.129	249 (87)	37 (13)	0.084
Yes	350 (82)	77 (18)		352 (82)	76 (18)	
**Continuity of Medical Care**	(2.50 ± 0.64)	(2.47 ± 0.70)	0.679	(2.51 ± 0.64)	(2.41 ± 0.73)	0.119
**Self-rated Health Status**	**(3.05 ± 1.00)**	**(3.45 ± 1.01)**	**0.000**	**(3.07 ± 0.98)**	**(3.38 ± 1.12)**	**0.007**
**HMO Membership**						
No	384 (84)	75 (16)	0.981	379 (83)	80 (17)	0.115
Yes	211 (84)	41 (16)		222 (87)	33 (13)	
**Sleeping disorder/Insomnia**						
No	**447 (86)**	**74 (14)**	**0.010**	**459 (87)**	**66 (13)**	**0.000**
Yes	**146 (78)**	**42 (22)**		**140 (75)**	**47 (25)**	
**Arthritis**						
No	**220 (93)**	**16 (07)**	**0.000**	**214 (90)**	**24 (10)**	**0.003**
Yes	**373 (79)**	**100 (21)**		**385 (81)**	**89 (19)**	
**Migraine Headache**						
No	489 (85)	87 (15)	0.06	**507 (88)**	**72 (12)**	**0.000**
Yes	104 (78)	29 (21)		**92 (69)**	**41 (31)**	
**Back pain**						
No	**346 (92)**	**32 (08)**	**0.000**	**344 (91)**	**34 (9)**	**0.000**
Yes	**247 (75)**	**84 (25)**		**255 (76)**	**79 (24)**	
**Level of Pain**	**(1.78 ± 2.14)**	**(3.11 ± 2.41)**	**0.000**	**(1.71 ± 2.01)**	**(3.25 ± 2.72)**	**0.000**
**Depressive Symptoms**	**(2.31 ± 2.69)**	**(2.86 ± 2.97)**	**0.047**	**(2.07 ± 2.44)**	**(4.16 ± 3.48)**	**0.000**
**Major Chronic Conditions**	**(2.32 ± 1.29)**	**(2.83 ± 1.30)**	**0.000**	**(2.32 ± 1.26)**	**(2.82 ± 1.46)**	**0.001**

**Table 3:** Multivariate logistic regression of opioid-based and psychotropic medication use among underserved African-American older adults (n = 740).

Independent Variables	Opioid-Based Medications			Psychotropic Medications		
	OR	95% CI	Sig.	OR	95% CI	Sig.
**Gender**						
Male	1.381	0.840–2.271	0.204	1.127	0.691–1.839	0.631
Female	1.00			1.00		
**Age**	1.013	0.985–1.042	0.382	0.997	0.968–1.028	0.861
**Education**	0.984	0.892–1.085	0.742	1.093	0.965–1.236	0.161
**Living Alone**						
No	1.137	0.718–1.799	0.584	1.147	0.712–1.848	0.572
Yes	1.00			1.00		
**Financial Strains**	0.856	0.685–1.070	0.172	1.189	0.967–1.461	0.1
**Continuity of Medical Care**	0.941	0.680–1.302	0.712	0.96	0.692–1.331	0.805
**HMO Membership**						
No	0.965	0.613–1.520	0.878	0.632	0.389–1.030	0.065
Yes	1.00			1.00		
**Self-rated Health Status**	**1.306**	**1.016–1.680**	**0.037**	0.876	0.687–1.118	0.288
**Sleeping disorder/Insomnia**						
No	1.097	0.676–1.782	0.707	1.125	0.693–1.828	0.633
Yes	1.00			1.00		
**Arthritis**						
No	**2.087**	**1.148–3.795**	**0.016**	1.309	0.755–2.270	0.337
Yes	**1.00**			1.00		
**Migraine Headache**						
No	0.977	0.577–1.655	0.932	1.634	0.984–2.714	0.058
Yes	1.00			1.00		
**Back pain**						
No	**2.206**	**1.338–3.638**	**0.002**	1.497	0.896–2.503	0.124
Yes	**1.00**			1.00		
**Level of Pain**	**1.122**	**1.006–1.251**	**0.038**	**1.126**	**1.008–1.258**	**0.036**
**Depressive Symptoms**	0.965	0.881–1.056	0.436	**1.143**	**1.053–1.241**	**0.001**
**Major Chronic Conditions**	1.139	0.970–1.336	0.112	1.123	0.952–1.325	0.17
**−2 Log Likelihood**	**557.659**			**531.068**		
**Nagelkerke**	**0.161**			**0.2**		
